# The Potential of Digital Solutions for Family Caregivers of Older Adults

**DOI:** 10.1093/geroni/igaf122.1136

**Published:** 2025-12-31

**Authors:** Sara Czaja

**Affiliations:** Weill Cornell Medicine, New York, New York, United States

## Abstract

Informal caregivers, such as family members provide the bulk of support to older adults with a chronic condition such as Alzheimer’s Disease or a Related Dementia (ADRD) and often do so at considerable cost to themselves. The roles of family caregivers are complex and range from assistance with daily activities and providing direct care to the care recipient to navigating complex health care and social services systems. As such, numerous studies have documented that ADRD caregiving has deleterious impacts on caregiver health and well-being. Unfortunately, for a variety of reasons, many caregivers do not avail themselves of existing services and programs and few evidenced-based programs are implemented in community settings. Digital technology solutions such as mobile applications, web-based platforms, sensing systems, and wearables hold promise in terms of enhancing the ability of caregivers to provide care and in reducing caregiver burden and distress. This presentation will discuss the potential of digital technology in providing support for family caregivers. An overview of available technology solutions will be provided with case exemplars. Additionally, data will be presented from on-going research projects regarding the feasibility and acceptability of digitally based solutions such as virtually delivered interventions, which overall show that caregivers are receptive to and able to use technology. The data from these studies also show that technology-based interventions are efficacious and improve caregiver outcomes. Finally, the potential benefits and challenges with these solutions, factors associated with the adoption of technology and recommendations for needed research in this area will also be discussed.

